# Optimizing duodenal tissue acquisition for mechanistic studies of duodenal ablation in type 2 diabetes

**DOI:** 10.1055/a-2503-2135

**Published:** 2025-01-29

**Authors:** Celine B.E. Busch, Kim van den Hoek, E. Andra Neefjes-Borst, Max Nieuwdorp, Annieke C.G. van Baar, Jacques J.H.G.M. Bergman

**Affiliations:** 1 1209 Gastroenterology and Hepatology, Amsterdam UMC, Location VUmc, Amsterdam, Netherlands; 2 1209 Pathology, Amsterdam UMC Location VUmc, Amsterdam, Netherlands; 3 26066 Internal and Vascular Medicine, Amsterdam UMC Location AMC, Amsterdam, Netherlands

**Keywords:** Endoscopy Upper GI Tract, Endoscopic resection (ESD, EMRc, ...), RFA and ablative methods, Endoscopy Small Bowel

## Abstract

**Background and study aims:**

Histological analysis of regular duodenal biopsies to study morphologic changes after duodenal ablation for type 2 diabetes (T2D) and metabolic syndrome is hampered by variability in tissue orientation. We designed an optimized tissue acquisition protocol using duodenal cold snare resections to create tissue microarrays (TMAs) and to allow for single-cell RNA sequencing (scRNA-seq).

**Patients and methods:**

The open-label DIRECT study included patients undergoing an upper gastrointestinal interventional endoscopy for non-duodenal indications. All underwent one ot two single-piece duodenal cold snare resections. Endpoints were safety, adequate histological orientation of specimen and TMA, and tissue dissociation quality for scRNA-seq. The optimized tissue acquisition protocol was validated in a duodenal ablation study, EMINENT-2.

**Results:**

In DIRECT, nine patients were included in whom a total of 16 cold snare resections were obtained. No severe adverse events (SAEs) occurred. Eighty percent of specimens and corresponding TMAs showed optimal tissue orientation. Further improvement was achieved by reducing tissue damage during endoscopic retrieval and improving histologic evaluation by eliminating ink use and pinning the tissue on cork. High-quality tissue dissociation scores for scRNA-seq were achieved in 13 of 18 samples (72%). In EMINENT-2, 38 cold snares were obtained without SAEs, histopathologic analysis showed good orientation in all samples, and dissociation scores for scRNA-seq were qualified in 35/38 (92%) samples.

**Conclusions:**

Duodenal cold snare resection is safe and can provide high-quality tissue for optimally oriented TMAs and high-quality tissue dissociation scores for scRNA-seq (Clinicaltrials.gov, NCT06333093, NCT05984238). This approach will allow mechanistic studies about the effects of duodenal ablation on metabolic syndrome and T2D.

## Introduction

Endoscopic duodenal ablation for treatment of type 2 diabetes (T2D) has emerged in recent years. Several techniques, including the Revita duodenal mucosal resurfacing (DMR) and recellularization via electroporation therapy (ReCET), have been shown in a number of clinical trials to have a beneficial effect on glycemic control and improvement in metabolic parameters in patients with T2D 1 2 3 . The hypothesis is that duodenal ablation improves insulin sensitivity. Mechanistic assessments have looked at incretins, bile acids, and the gut microbiome 4 5 6 7 . However, the mechanism of action is still largely unknown.

**Fig. 1:**
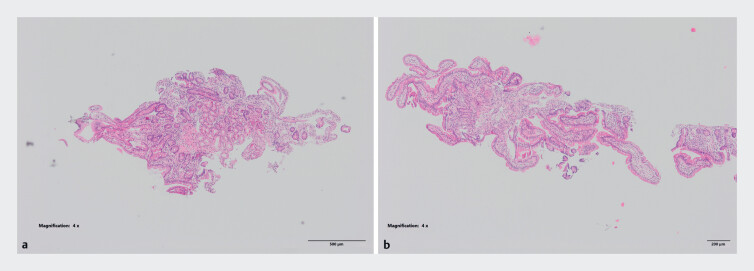
H&E slides of regular biopsies from one patient from the INSPIRE study. H&E, hematoxylin and eosin.

The endoscopic appearance and histology of biopsy samples taken before and after DMR and ReCET suggest a normal duodenal mucosa. However, histomorphologic changes in duodenal mucosa after the procedures have not been extensively studied. This is partly due to the limited scope for obtaining duodenal biopsies in most human studies, due to concerns about influencing the clinical course after ablation in any way. The INSPIRE study 2 (EudraCT 2017–00349–30) was the first duodenal ablation study in which duodenal biopsies were taken before and after DMR, yet the study was hampered by significant variability in tissue orientation between biopsies, even for biopsies that were taken during the same endoscopic procedure under identical conditions. This variability is probably caused by tangential orientation of the biopsy forceps onto the tissue, immediate curling of the tissue upon immersion in formalin, random orientation of the specimens upon embedding of the tissue in paraffin, and subsequent tangential sectioning of the paraffin blocks ( Fig. 1 ). As a result, quantitative assessment of duodenal mucosa and submucosa, with measurements such as villus length and crypt depth, is overwhelmed by background variability in tissue orientation. The above challenges persisted during the EMINENT study (International Clinical Trials Registry Platform, NL9482), an open-label study of ReCET in patients with T2D 1 .

As the evidence on safety, feasibility and efficacy of duodenal ablation techniques continues to grow, understanding how these techniques affect glycemic and metabolic control has become even more important. For such mechanistic studies, optimal tissue acquisition before and after ablation is a prerequisite, especially because our group has initiated two sham-controlled randomized duodenal ablation trials (EMINENT-2 study [NCT05984238] and REMIND study [NCT06092476]) in which, for the first time, mechanistic studies have been incorporated. Both studies aim to evaluate changes before and after duodenal ablation compared with a sham procedure using single-cell ribonucleic acid sequencing (scRNA-seq) and optimally oriented tissue microarrays (TMAs).

In recent years, cold snare polypectomy has become common practice for removal of sessile polyps in the colon 8 . Compared with standard electrocoagulation polypectomy, this procedure is safer, easier, and faster 9 10 . A cohort study indicates that larger piecemeal cold snare resections are feasible in the colorectum with a negligible risk of post-resection bleeding and perforation 11 . The technique has subsequently been used in the duodenum for removal of sessile polyps and piecemeal resections of larger, benign-looking flat polyps 12 13 .

In the INSPIRE study, we obtained 12 biopsies before and after DMR in all patients. Based on the safety profile of cold snare resections, we hypothesized that performing one to two cold snare resections would be as safe as the extensive biopsy protocol of the INSPIRE study, and would provide more suitable material for scRNA-seq and would allow for optimally oriented TMAs.

In preparation for the two aforementioned sham-controlled randomized trials, we developed and tested a cold snare-based duodenal tissue acquisition protocol in an independent cohort. This article systematically outlines our approach, placing emphasis on the safety, feasibility, and potential benefits of this novel duodenal sampling method, resulting in a standardized tissue acquisition protocol. We also present safety and feasibility results from cold snaring in the sham-controlled EMINENT-2 study.

## Patients and methods

### Initial endoscopic cold snare resection technique

All cold snare resections were obtained by a single endoscopist (JB) who also performs all endoscopic duodenal ablation procedures in the Netherlands. A pediatric colonoscope (PCF-H190TL; Olympus Corporation, Tokio, Japan) was used in order to reach the horizontal part of the duodenum. Saline (sodium chloride 0.9%) stained with methylene blue (methylammonium chloride 5 mg/mL) was drawn into 10-mL syringes. A 23-gauge injection therapy needle (Interject; Boston Scientific, Marlborough, Massachusetts, United States) was advanced through the working channel of the scope and directed to the selected site in the duodenum. Subsequent to submucosal needle insertion, 3 to 5 mL of “blue saline” was injected. A 15-mm cold snare (Diamond Cut; Micro-Tech Endoscopy, Nanjing, China) was then advanced into the duodenum, positioned around the elevated duodenal mucosa, and closed for cold snare cutting without forcefully pulling the snare back in the scope. Closure of the mucosal defect was secured by placing one to two hemoclips (Resolution 360 Clip; Boston Scientific, Marlborough, Massachusetts, United States). The excised tissue was aspirated through the endoscope working channel and collected in a collection box. A small pea-sized piece was cut from the specimen, rinsed three times with phosphate-buffered saline, and placed in a tube containing sCelLiVE Tissue Preservation Buffer (Singleron Biotechnologies, Cologne, Germany). The tissue preservation buffer stimulates the physiological environment and ensures high cell viability over a 72-hour period. The tissue-filled tube was sent to Singleron Biotechnologies for tissue dissociation using the Singleron PythoN Automated Tissue Dissociation System. A detailed description of the tissue dissociation process at Singleron Biotechnologies can be found in **Supplementary Material A** . The remaining tissue (approximately 10 mm in length) was directly placed into a cassette with an open-celled foam sponge pad and manually spread out by one of the investigators. The cassette was then immersed in 4% buffered formalin for 24 to 48 hours and subsequently embedded in paraffin, resulting in a formalin-fixed paraffin embedded (FFPE) block. The samples were embedded on edge, with the mucosal side facing sideways, to allow cutting of the specimen perpendicular to the mucosal surface, creating a full mucosal thickness specimen with optimal tissue orientation. Hematoxylin and eosin (H&E)-stained slides were obtained and evaluated by an expert pathologist, who identified the part where the slide portrayed the tissue most accurately. Next, a 2.5-mm core was punched out from these sections, which were used to make a TMA by Applied Biological Materials Inc. (Richmond, Canada).

### DIRECT study: Design, participants, and endpoints

The open-label DIRECT study was initiated to test the envisioned tissue sampling and tissue handling protocol outside clinical duodenal ablation studies. This study was conducted at the Amsterdam UMC between January 2023 and May 2023. The study was approved by the Medical Ethics Committee of the Amsterdam University Medical Centers (Amsterdam UMC) and registered as NCT06333093 at Clinicaltrials.gov.

Patients were eligible if they were scheduled for an upper gastric or esophageal interventional endoscopy under deep sedation with propofol at the Amsterdam UMC. Exclusion criteria included a history of gastrointestinal surgery affecting gastrointestinal anatomy and impeding endoscopic access to the duodenum (e.g. Roux-en-Y gastric bypass) and a history of duodenal inflammatory diseases (e.g. Crohn’s disease and celiac disease). Written informed consent was obtained from all patients prior to endoscopy.

The primary study endpoints were safety and feasibility. The primary safety endpoint was incidence of serious adverse events (SAEs) after cold snare resections. Feasibility was evaluated based on ability to acquire adequate histological samples, including endoscopic acquisition of duodenal tissue and fixation, slicing, and staining of samples. Secondary endpoints included ability to obtain adequate orientation in the tissue slides where adequate assessment of villus and crypt length, thickness of the different tissue layers, and cell densities would be possible at pathologist discretion, feasibility of creating a TMA, and tissue dissociation quality assessment results from Singleron Biotechnologies as a quality measure for future scRNA-seq. Tissue dissociation quality control results were based on the standard quality criteria from Singleron Biotechnologies as shown in Table 1 .

**Table:** **Table 1** Singleron Biotechnologies standard quality criteria for tissue dissociation.

**Grade**	**Cell number (cells)**	**Cell viability (%)**
A (qualified)	≥ 20,000	≥ 85
B (suboptimal)	≥ 20,000	≥ 70
C (unqualified)	< 20,000	< 70

#### Method evaluation and interim analysis

During the course of the DIRECT study, several optimizations of the initial endoscopic cold snare resection technique were implemented. An interim analysis was conducted in collaboration with the involved pathologist (ANB) after obtaining and processing four tissue resection specimens. After each additional analysis, adjustments were made to improve tissue quality in consultation with the involved endoscopist. The main modifications are outlined in the following section.

#### Implemented optimization steps for endoscopic cold snare resections and tissue handling

1. Minimization of tissue curling using pinning on cork. Persisted tissue curling at the edges of the tissue samples was observed when using cassettes with sponge foam pads. Inspired by preparation of endoscopic submucosal dissection specimens, tissue samples were pinned on cork. Careful stretching of the tissue, without overstretching, was performed using pins at the lateral sides of the specimens 14 .
2. Mitigation of tissue damage using a basket for endoscopic retrieval. Aspiration of resected tissue specimen through the working channel of the endoscope, as was the general approach to retrieve cold snare resection specimens for other indications in our unit, resulted in significant tissue damage and fragmentation. To address this, the resected specimens were delicately captured using a 3-cm retrieval basket (Roth net, Steris, Mentor, Ohio, United States). The net, along with the tissue and the endoscope, were subsequently extracted from the patient. Initially, wooden sticks were used to remove tissue from the biopsy collector, followed by manual spreading the tissue on cork. This was replaced by removing the specimens from the net by a pair of tweezers, placing them on cork, and carefully spreading them using pins.
3. Optimization of pathology assessment by refraining from using ink. We eliminated routine application of Indian ink at the deeper margin of the resection. We found this to be a disturbing factor in histopathologic analysis. Ink usually facilitates assessment of whether the specimen has clean, i.e. non-neoplastic resection margins. However, this was not applicable for our research purposes.

### EMINENT-2 study: Design, participants and endpoints

The optimized protocol resulting from the initial DIRECT study was subsequently validated in our sham-controlled randomized trial, the EMINENT-2 study, which was approved by the Medical Ethics Committee of the Amsterdam UMC. Enrollment began in July 2023 and is still ongoing. Written informed consent was obtained from all patients.

Inclusion criteria were T2D, age between 28 and 75 years, body mass index (BMI) 24 to 40 kg/m², maximum hemoglobin A1c (HbA1c) of 8.0% (64 mmol/mol), adequate beta-cell reserve (fasting C-peptide > 0.2 nmol/L), and use of basal insulin. Exclusion criteria were type 1 diabetes, history of ketoacidosis, and use of short-acting insulin or a glucagon-like peptide-1 receptor agonist (GLP-1RA). Patients were randomized to receive either the ReCET (Endogenex Inc.; Plymouth, Minnesota, United States) or a sham procedure, followed by a 12-week follow-up endoscopy. Unblinding occurred at 24 weeks, after which eligible patients crossed over, following another follow-up endoscopy after 12 weeks. During all endoscopies a cold snare resection was obtained. Primary endpoints and clinical outcomes will be described in a separate manuscript. Cold snare resections were obtained according to the tissue acquisition protocol developed in the DIRECT study, using the same outcome parameters as described for the DIRECT study.

## Results

### DIRECT study: Baseline characteristics and primary endpoints

Nine patients were included with a median age of 65 years (interquartile range [IQR] 60–71) and median BMI of 22.7 kg/m ^2^ (IQR 21.3–25.9). Two cold snare resections were obtained from seven patients and one cold snare resection from two patients, totaling 16 samples. No post-procedure SAEs or AEs were observed.

### DIRECT study: Secondary endpoints

**Fig. 2:**
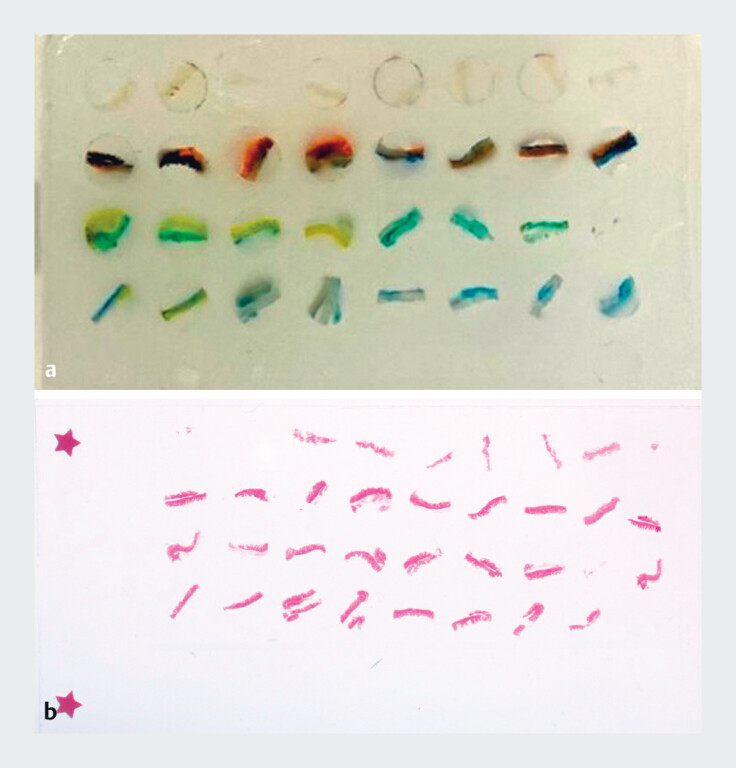
**a** Macroscopic picture of formalin-fixed paraffin embedded tissue microarray block. **b** Macroscopic picture of H&E-stained slide of the tissue microarray. H&E, hematoxylin and eosin.

**Fig. 3:**
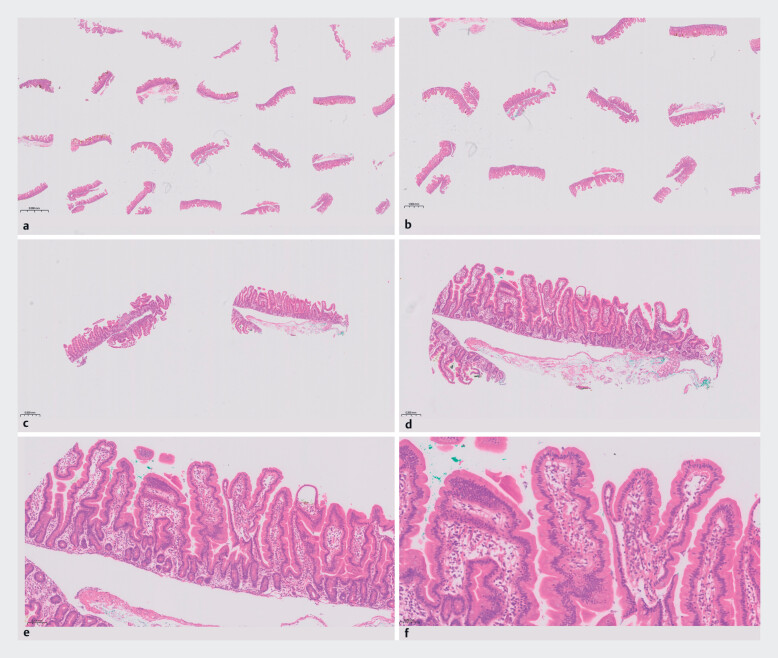
**a** Microscopic images of H&E-stained slide of tissue microarray. **b** 1x magnification. **c** 2x magnification. **d** 5x magnification. **e** 10x magnification. **f** 20x magnification. H&E, hematoxylin and eosin.

Histopathologic assessments were performed after every four tissue resections. After obtaining 12 samples, the pathologist was satisfied with orientation of the samples. The tissue could be adequately assessed, at least 80% of the specimen cuts showed optimal orientation and allowed reliable measurements of the length of villi and crypts, thickness of the mucosal and submucosal layers, and cell density. The FFPE samples of tissue also enabled creation of well-oriented TMAs ( Fig. 2 and Fig. 3 ).

Eighteen pea-sized fresh tissue samples were obtained from eight patients, all of which were sent to Singleron Biotechnologies. Thirteen samples were graded as A (72% of total), one sample was graded B (6% of total), and two samples were graded C (11% of total). Two samples could not be assessed due to delayed transport. After several consecutive A-graded samples, Singleron Biotechnologies indicated that further optimization was not necessary. A detailed overview of the quality scores per sample is show in **Supplementary Table 1** .

### Standardized method to obtain endoscopic large cold snare duodenal mucosectomies

**Fig. 4:**
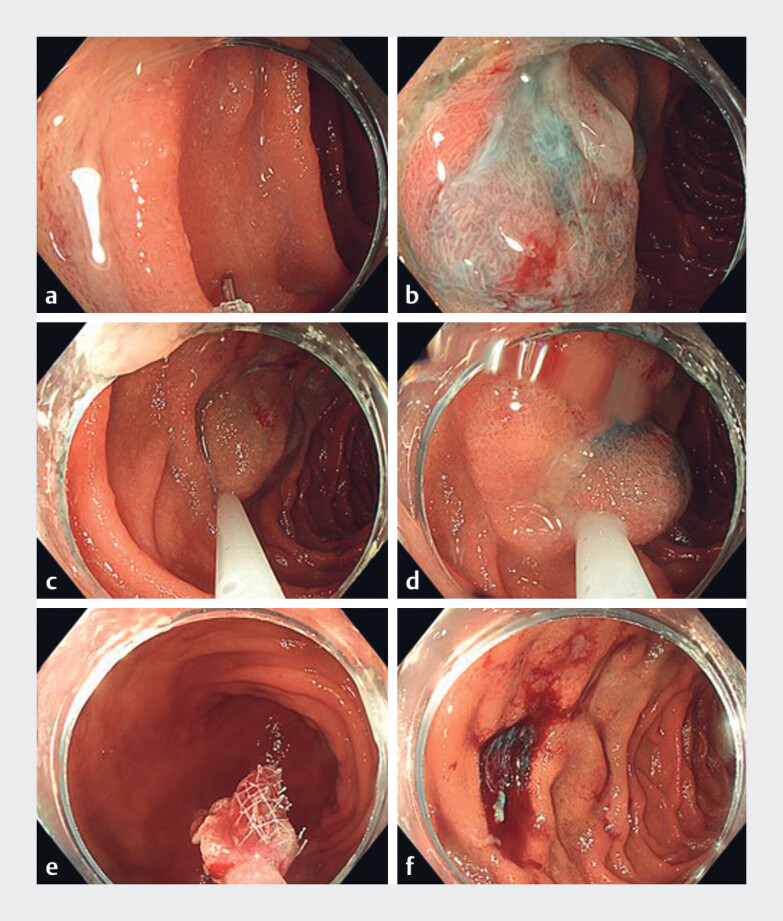
Endoscopic photos of the acquiring of a cold snare resection in the duodenum. **a** Normal-looking duodenum and the injection needle. **b** Moments after injection of submucosal saline. **c** Cold snare is placed around the lifted tissue. **d** Closure of the snare. **e** The tissue is gently held with the Roth net. **f** The duodenum moments after the mucosectomy has been obtained.

**Fig. 5:**
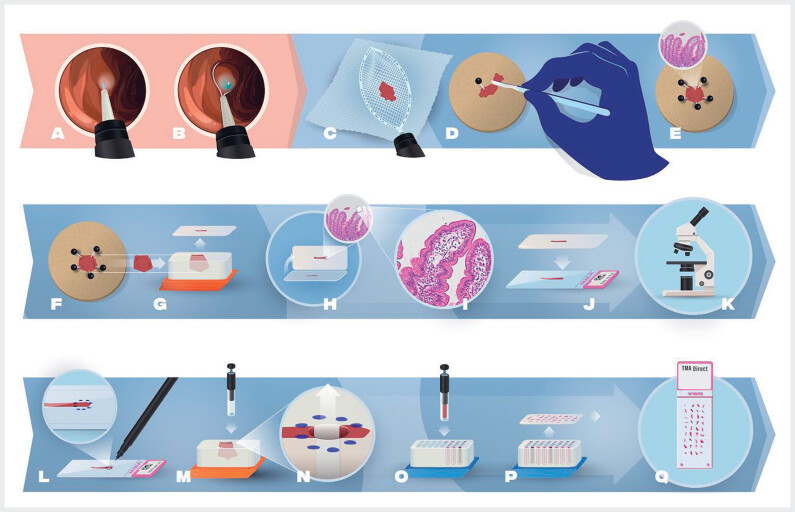
Step-by-step explanation of duodenal tissue processing. **a** Inject blue saline into the submucosa, causing tissue elevation. **b** Position the snare over the elevated tissue and close the snare to excise the tissue using standard cold snare polypectomy technique. **c** Retract the piece of tissue with a Roth net. **d** Transfer the tissue to a cork using tweezers and cut a pea-size tissue sample for single-cell RNA sequencing (optional). **e** Use additional pins to spread the (remaining) tissue without overstretching. **f,g** Have the tissue embedded on edge. **h,i,j** Create an H&E-stained slide of each FFPE block. **k,l** Identify the most representative area of the mucosectomy and have it marked. **m,n** Extract an appropriate core from the embedded specimen with a microarrayer and use the markings on the H&E slide as a reference. **o** Place the tissue core into the recipient FFPE TMA block. **p,q** Create slides of the TMA and apply the desired stains for additional histopathologic analyses. FFPE, formalin-fixed paraffin-embedded; H&E, hematoxylin and eosin; RNA, ribonucleic acid; TMA, tissue microarray.

Findings from the DIRECT study resulted in the following advised method of acquiring cold snare resections as shown endoscopically in Fig. 4 and graphically in Fig. 5 .

1. Advancement of the pediatric colonoscope into the duodenum and selection of the site for obtaining the mucosectomy.
2. Insertion of the injection needle through the working channel of the endoscope and injection of a sufficient amount of blue saline into the submucosa, causing tissue elevation and bluish discoloration ( Fig. 5 **a** ).
3. Advancement of the 15-mm cold snare through the scope, opening of the snare, and positioning of it over the elevated tissue, and closure of the snare to excise the tissue using standard cold snare polypectomy technique ( Fig. 5 **b** ).
4. Replacement of the cold snare with a Roth net and gentle movement of the fully opened net over the resection site to separate the specimen from the surrounding mucosa. Minimal closure of the net to avoid any mechanical damage to the specimen. Closure of the net over the tissue sample without excessive compression. Withdrawal of the net with the entrapped tissue and the endoscope from the patient ( Fig. 5 **c** ).
5. Reintroduction of the endoscope and inspection of the wound bed after resection. If necessary, placement of an (prophylactic) endoscopic clip.
6. Careful transfer of the tissue from the opened net to a cork using tweezers and securement of the tissue with one pin ( Fig. 5 **d** ). The luminal (or villous) side should be the upper side, the submucosa on the cork. A 10x hand-held magnifying glass is used to visualize the villous side.
7. If desired, a pea-size tissue sample is cut for single-cell RNA sequencing (optional) ( Fig. 5 **d** ).
8. Use of additional pins to spread the (remaining) tissue without overstretching ( Fig. 5 **e** ) and placement of the pinned tissue upside down in 4% buffered formalin.
9. Tissue embedding on edge ( Fig. 5 **f** and Fig. 5 **g** ). If the tissue is too big to be embedded en-bloc, it should be sliced into two or more fragments, always perpendicular to the surface.
10. Creation of a H&E-stained slide of each FFPE block ( Fig. 5 **h** , Fig. 5 **i** , Fig. 5 **j** ), identifying the most representative area of the mucosectomy and marking it ( Fig. 5 **k** and Fig. 5 **l** ).
11. Extraction of an appropriate core from the embedded specimen using a microarrayer and the markings on the H&E slide as a reference ( Fig. 5 **m** and Fig. 5 **n** ). Placement of the tissue core into the recipient FFPE TMA block ( Fig. 5 **o** ).
12. Repetition of this process with other cold snare resections (from other patients and/or other time points of the same patient) until the desired number of tissue pieces is are in the recipient FFPE TMA block ( Fig. 5 **p** ).
13. Cutting of sections of the TMA and application of the desired stains for additional histopathologic analyses ( Fig. 5 **q** ).

### EMINENT-2: Baseline characteristics and endpoints

Twenty-one patients were included with a median age of 66 years (IQR 60–68) and median BMI of 27.5 kg/m ^2^ (IQR 26.6–30.1). To date, 16 patients have undergone 12-week follow-up endoscopy and one patient received the cross-over procedure. A total of 38 cold snare resections were performed. No post-procedural SAEs or AEs were observed.

Tissue from 21 patients at different timepoints was sent for tissue dissociation and subsequent scRNA-seq. Thirty-five samples were graded A (92% of total), three samples were graded B (8% of total), and no samples were graded C. **Supplementary Table 2** is a detailed overview of the quality after tissue dissociation.

## Discussion

These studies are the first to report use of cold snare resections in healthy duodenal mucosa for scientific studies. We did not observe any AEs or SAEs in more than 50 patients, and combined with the reported safety profile of cold snare resections in colon and duodenum, we can conclude that our stepwise approach to acquiring duodenal tissue via a cold snare resection appears safe.

We designed an optimized protocol for tissue resection, specimen retrieval, handling and processing for research purposes and provide a detailed description with corresponding illustrations. This method enables adequate and reliable histopathologic analysis, both by traditional histopathologic assessment of tissue and by immunohistochemical staining of a TMA, overcoming limitations of using regular duodenal biopsies for this purpose. In addition, it provides a larger piece of tissue for a variety of other assays such as scRNA-seq. In our opinion, our protocol will enable better mechanistic studies into the effect of duodenal ablation in patients with T2D and metabolic syndrome. It may also find a wider application for other basic and translational studies into the function of the gastrointestinal tract. We used a 15-mm snare for our cold snare resections but avoided full 15-mm snare resections of duodenal mucosa, and did not use the technique of forcefully pulling the closed snare to the tip of the endoscope. We feel that this increases risk of complications and mechanically damages the resected specimen. In retrospect, a 10-mm snare might be more appropriate for this purpose, which is in agreement with the recent European Society of Gastrointestinal Endoscopy (ESGE) guideline 15 .

Our study has several limitations. First, the study had a relatively small sample size. However, this was an optimization study and after 10 patients, it was decided that the optimal protocol had been achieved. Second, alternative techniques for obtaining duodenal tissue were not explored because the study focused on improving acquisition of duodenal tissue using the cold snare resection technique. Third, the generalizability of our results has not yet been demonstrated, because all procedures were performed by a single endoscopist. However, given the detailed description of our method, and the widespread use of cold snare resections in gastrointestinal endoscopy, we are convinced this method can be easily performed by other endoscopists.

Results of the initial DIRECT study were validated in our sham-controlled randomized trial, EMINENT-2. The optimized and standardized protocol improved homogeneity of tissue acquisition and processing. This will ultimately lead to reliable histopathologic and cellular analyses comparing tissue before and after ablation in EMINENT-2 and REMIND. This advancement is expected to enhance our understanding of the mechanism of action of duodenal ablation in patients with T2D.

## Conclusions

In conclusion, performing cold snare resections to retrieve small-sized mucosectomies for research purposes in the duodenum using a 15-mm cold snare appears safe and feasible. For widespread use, we recommend using a 10-mm cold snare following the recent ESGE guideline. Cold snare resections are a better alternative to multiple regular biopsies for both histological and cellular activity research purposes.

## Supplementary Material

Supplementary Material Zusatzmaterial
