# Supplementary material for: Optimizing duodenal tissue acquisition for mechanistic studies of duodenal ablation in type 2 diabetes
Source: Endosc Int Open. 2025 Jan 29;13:a25032135. doi: 10.1055/a-2503-2135 (PMC11855243; doi:10.1055/a-2503-2135)
Supplement: Supplementary file 1 — Supplementary Material [file 10-1055-a-2503-2135_25190774.pdf]

SUPPLEMENTARY MATERIAL

Supplementary material A

Tissue dissociation process Singleron Biotechnologies

Dissociation is performed using the Singleron PythoN Automated Tissue Dissociation System. The tissue section is washed 3 times in 1x HBSS (Hank's Balanced Saline Solution, Gibco, REF Nr.: 24020-117) and placed in a 2-mL Eppendorf tube (Eppendorf, Hamburg, Germany) and weighed. The tissue is then briefly cut with ophthalmic scissors in 500 µL of sCelLiVE Tissue Dissociation Buffer and transferred to the Singleron PythoN Dissociation Unit (DNFU). The final volume of the dissociation mixture is calculated according to the amount of tissue. In detail, 500 µL, 1000 µL, or 2000 µL of dissociation solution is used for up to 50 mg, 100 mg, or more than 100 mg of tissue, respectively. The automated dissociation is performed at 180 rpm, including 36 cycles of 15 CCW and 10 CW rotations. The sample is incubated at 37°C during the whole program (15 min). Afterward, the cell suspension is filtered using a 40-µm sterile strainer (EASYstrainer, Greiner Bio-one, REF Nr.: 542040). The filtered suspension is washed twice with phosphate-buffered saline by centrifugation at 4°C, 350 g for 5 minutes. The resulting pellet containing single cells is resuspended in 1000 µL cold PBS. Cells are counted using acridine orange/propidium iodide double staining with Luna FX7 automated cell counter (Logos Biosystems, Villeneuve d'Ascq, France). Tissue dissociation quality control results are based on the standard quality criteria of Singleron Biotechnologies. If necessary, removal of erythrocytes, dead cells, and/or debris is performed according to Singleron Biotechnologies protocols.

Endosc Int Open | DOI 10.1055/a-2503-2135 | © The Author(s).

Supplementary Table 1 Quality report after tissue dissociation of tissue obtained in the DIRECT study sent for single-cell RNA sequencing.

Sample	Date	Tissue weight (mg)	Cell number	Cell viability (%)	Quality score
Di001A	12-1-2023	< 1 mg	19,080	83.3	C
Di001B	12-1-2023	< 1 mg	108,000	95.6	A
Di002A	26-1-2023	31	142,000	93.9	A
Di002B	26-1-2023	54	122,060	98.9	A
Di002C	26-1-2023	42	74,290	97.5	A
Di002D	26-1-2023	17	1,219	97.5	C
Di003A	7-2-2023	30	417,000	91.0	A
Di003B	7-2-2023	42	1,720,000	97.5	A
Di003C	7-2-2023	10	33,000	79.8	B
Di005A	18-4-2023	30	1,140,000	98.3	A
Di005B	18-4-2023	29	3,200,000	99.1	A
Di006A	3-4-2023	49	NA*	NA	NA
Di006B	3-4-2023	NA	NA*	NA	NA
Di007A	16-5-2023	83	3,328,000	97.7	A
Di007B	16-5-2023	17	1,375,000	98.1	A
Di008A	16-5-2023	40	2,685,000	97.6	A
Di008B	16-5-2023	10	595,000	98.9	A
Di009A	23-5-2023	20	1,770,000	97.3	A

*Due to delayed transport, the temperature of the tissue was too high to get reliable results.

Endosc Int Open | DOI 10.1055/a-2503-2135 | © The Author(s).

Supplementary Table 2 Quality report after tissue dissociation of tissue obtained in the EMINENT-2 study sent for single-cell RNA sequencing.

Sample	Timepoint	Date	Tissue weight (mg)	Cell number	Cell viability (%)	Quality score
EM2-002	T0	21-08-2023	40	7.200.000	93.0	A
EM2-004	T0	21-08-2023	40	8.770.000	94.9	A
EM2-001	T0	25-09-2023	10	2.350.000	93.9	A
EM2-003	T0	25-09-2023	10	948.000	89.9	A
EM2-005	T0	25-09-2023	50	1.690.000	83.8	A
EM2-006	T0	25-09-2023	20	1.820.000	89.1	A
EM2-002	T12W	22-11-2023	70	556.000	97.1	A
EM2-004	T12W	22-11-2023	100	118.600	94.6	A
EM2-010	T0	22-11-2023	20	61.200	99.2	A
EM2-011	T0	22-11-2023	50	471.500	95.3	A
EM2-012	T0	22-11-2023	60	765.000	94.6	A
EM2-003	T12W	13-12-2023	70	3.460.000	87.3	A
EM2-005	T12W	13-12-2023	40	8.760.000	89.5	A
EM2-006	T12W	13-12-2023	30	3.360.000	97.3	A
EM2-001	T12W	19-12-2023	23	795.000	93.3	A
EM2-008	T0	19-12-2023	114	1.180.000	93.8	A

Endosc Int Open | DOI 10.1055/a-2503-2135 | © The Author(s).

EM2-009	T0	19-12-2023	104	2.980.000	75.9	B
EM2-013	T0	19-12-2023	60	745.000	91	A
EM2-015	T0	31-01-2024	< 10	2.583.000	94.8	A
EM2-016	T0	31-01-2024	< 10	870.000	90.9	A
EM2-014	T0	12-02-2024	60	2.660.000	76.2	B
EM2-017	T0	12-02-2024	65	1.790.000	92.2	A
EM2-010	T12W	14-02-2024	50	2.510.000	97.4	A
EM2-011	T12W	14-02-2024	50	2.510.000	87.6	A
EM2-012	T12W	14-02-2024	30	2.260.000	94.2	A
EM2-008	T12W	18-03-2024	30	984.000	86.4	A
EM2-009	T12W	18-03-2024	30	2.292.000	88.1	A
EM2-013	T12W	18-03-2024	30	882.000	71.3	B
EM2-019	T0	22-04-2024	80	1.460.000	96.4	A
EM2-023	T0	22-04-2024	40	478.000	97.0	A
EM2-016	T12W	22-04-2024	167	6.050.000	91.7	A
EM2-015	T12W	02-05-2024	50	1.100.000	86.1	A
EM2-014	T12W	08-05-2024	20	880.000	93.1	A
EM2-017	T12W	08-05-2024	31	1.030.000	99.2	A
EM2-022	T0	08-05-2024	40	880.000	93.1	A
EM2-025	T0	10-06-2024	72	1.935.000	98.2	A

Endosc Int Open | DOI 10.1055/a-2503-2135 | © The Author(s).

EM2-026	T0	10-06-2024	50	1.415.000	94.8	A
EM2-008	T0C	10-06-2024	70	1.455.000	97.3	A

RNA, ribonucleic acid.

Endosc Int Open | DOI 10.1055/a-2503-2135 | © The Author(s).
